# Risk stratification of IBD-associated liver disease using routinely collected biomarkers from a large-scale real-world dataset

**DOI:** 10.1136/bmjgast-2025-002028

**Published:** 2025-11-13

**Authors:** Zachary Green, Alex Z Kadhim, Lynn Win, Gabriela Czanner, Robert Mark Beattie, Sarah Ennis, James John Ashton

**Affiliations:** 1 Department of Human Genetics and Genomic Medicine, University of Southampton, Southampton, England, UK; 2 Department of Paediatric Gastroenterology, Southampton Children’s Hospital, Southampton, UK; 3 Department of Electronics and Computer Science, University of Southampton, Southampton, England, UK; 4 Southampton Biomedical Research Centre, NIHR, Southampton, England, UK

**Keywords:** IBD, Liver function test, Primary sclerosing cholangitis, Autoimmune biliary disease

## Abstract

**Objective:**

Inflammatory bowel disease (IBD)-associated liver diseases (IBDALDs) are associated with hepatobiliary damage and malignancy, with diagnosis often delayed by heterogeneous presentation. We evaluated whether routinely collected biomarkers—at IBD diagnosis and during follow-up—can risk-stratify for IBDALD.

**Methods:**

This observational retrospective longitudinal study included 1571 patients with IBD at University Hospital Southampton. Biomarkers including alanine aminotransferase (ALT), alkaline phosphatase (ALP) and erythrocyte sedimentation rate (ESR) (n=335 605 results) were summarised as patient-level medians within ±6 months of IBD diagnosis. Patients with pre-existing IBDALD were excluded. A 1:4 matched case-control design (age, sex, IBD subtype) was applied. Conditional logistic regression assessed associations with biomarkers (continuous values and binary—abnormal vs normal) and IBDALD. Longitudinal trends were evaluated using locally estimated scatterplot smoothing (LOESS) and linear mixed-effects models (LMMs).

**Results:**

Median age of IBD diagnosis was 18.0 years, median follow-up 11.5 years. Thirty-five IBDALD cases were identified (27 post-IBD); median time to IBDALD was 4.5 years. At IBD diagnosis, cases had elevated ALT, ALP and ESR (p<0.01). In case-control matching, ALT (OR=1.04 per U/L; 95% CI 1.01 to 1.07; p=0.012), ALP (OR=1.01; 95% CI 1.00 to 1.02; p=0.014) and ESR (OR=1.05; 95% CI 1.00 to 1.09; p=0.034) were associated with IBDALD. Any abnormal ALT (OR=5.10; 95% CI 1.57 to 16.59; p=0.0068) and ALP (OR=15.33; 95% CI 1.87 to 125.77; p=0.0110) were strongly associated. LOESS plots and LMMs demonstrated distinct biomarker trajectories (ALT, ALP) preceding IBDALD.

**Conclusion:**

Real-world biomarker data can support early risk stratification for IBDALD. Elevated ALT and ALP at IBD diagnosis and distinct longitudinal trajectories highlight the need for follow-up to biomarker normalisation, with persistent abnormalities prompting earlier hepatobiliary investigation to reduce diagnostic delay and improve outcomes.

**WHAT IS ALREADY KNOWN ON THIS TOPIC**

- Autoimmune liver diseases are serious and rare complications of inflammatory bowel disease (IBD).
- Diagnosis is often delayed due to non-specific early signs and lack of predictive biomarkers, culminating in poor clinical outcomes.

**WHAT THIS STUDY ADDS**

- This study demonstrates that patients with IBD, who later develop autoimmune liver disease, are more likely to have elevated liver biomarker profiles evident at the time of IBD diagnosis.
- Longitudinal analysis of routinely collected biomarkers reveals differing trajectories over time, supporting the potential for early risk stratification using real-world clinical data.

**HOW THIS STUDY MIGHT AFFECT RESEARCH, PRACTICE OR POLICY**

- This study suggests that early biomarker-based risk stratification may help identify patients with IBD at higher risk of developing autoimmune liver disease.
- These findings could inform closer monitoring, timely investigations and earlier treatment, with the potential to reduce diagnostic delays and improve outcomes.

## Introduction

 Inflammatory bowel disease (IBD) is a collection of chronic, relapsing and remitting conditions which can be divided into Crohn’s disease (CD), ulcerative colitis (UC) and IBD-unclassified (IBDU).^1^ Many extraintestinal manifestations (EIMs) of IBD are described, commonly affecting the joints, skin, bones, eyes, lungs, cardiovascular system and hepatobiliary tract.^2^ The predominant autoimmune hepatobiliary disorders associated with IBD (IBD-associated liver diseases, hereon collectively referred to as IBDALD) include primary sclerosing cholangitis (PSC), autoimmune hepatitis (AIH) and AIH-PSC ‘overlap syndrome’.^3^ These disorders carry significant morbidity through liver failure, portal hypertension and hepatic and colonic cancers.^4^

Identification of IBALD is challenging. Clinical presentation of these conditions is varying, from asymptomatic derangement in blood tests to acute liver failure. As many as one-third of patients with IBD have been noted to have deranged liver function tests, which are often transient; however, with only 5% developing liver disease.^5^ Abnormal liver function tests can also be secondary to multiple causes, including metabolic dysfunction-associated steatotic liver disease, IBD therapies, as in drug-related liver injury and infection.^3^ Early efforts to risk stratify for IBDALDs have shown promise. *Wang et al* devised a Polygenic Risk Score (PRS) for PSC and demonstrated that patients with IBD in the highest PRS quartile had over twice the risk of developing PSC (7.2%) compared with those in the lowest quartile (3.0%). However, this approach has not yet been clinically validated in independent cohorts.^6^

While no singularly definitive biomarker has been identified to predict individuals at risk of clinically significant sequelae in IBD, emerging evidence underscores the potential of integrating large-scale clinical and multiomic data for this purpose.^7^ Such approaches may incorporate ‘big data’ techniques to further improve risk stratification and support personalised clinical decision-making through the development of risk stratification tools.^8^ Longitudinal trends in multiomic, large-scale data have demonstrated utility for the study of IBD trajectories and to predict disease outcomes.^9^

This study aimed to leverage a large-scale, real-world clinical dataset to evaluate the risk of developing IBDALD. We aimed to characterise early biomarker signatures present at the time of IBD diagnosis as well as longitudinal patterns in biomarker levels preceding the onset of IBDALD to inform earlier disease detection.

## Methods

This was an observational retrospective longitudinal study. The cohort for this analysis was the IBD patient-only ‘Genetics of IBD’ study (09 /H0504/125, University of Southampton, UK). All individuals have a confirmed histological diagnosis of IBD according to Porto criteria or British Society of Gastroenterology guidance.^10 11^ Individuals in the cohort include children and young people diagnosed and managed by the Southampton Children’s Hospital (SCH) paediatric IBD service—the specialist referral centre for the Wessex region—and the adult IBD service at University Hospital Southampton (UHS). At the time of data extraction and analysis (November 2024), 1571 patients were enrolled.

### Data collection and curation

Data were extracted from electronic health records. These included demographic information (date of birth, gender), detailed IBD diagnosis data (diagnosis date, disease location and subtype: UC, CD or IBDU) and laboratory test results (including haematology and biochemistry). Anthropometric measures, such as height, weight, body mass index (BMI) and BMI SD scores (BMI-SDS), are routinely extracted. Comorbidity data are derived from coded diagnoses recorded in clinical inpatient discharge summaries. All laboratory results originate from routine clinical care and are therefore recorded at irregular, non-standardised intervals.

Routinely collected biomarkers analysed were liver function tests (alanine aminotransferase (ALT), alkaline phosphatase (ALP), total bilirubin (TB), aspartate aminotransferase (AST) and gamma-glutamyl transferase (GGT)), inflammatory markers (C-reactive protein (CRP) and erythrocyte sedimentation rate (ESR)), in addition to albumin (ALB). Each biomarker datapoint is associated with the reference range of the test (age and gender specific) and the range flag, which indicates whether the result is low, normal or high relating to this range.

A locally deployed large-language model (LLM) Llama 3.1 70B (llama3.1:8b-instruct-fp16) was used to identify cases of IBDALD within the total cohort from redacted histology and radiology records.^12^ The output of this model was manually verified (ZG) against the electronic clinical documents as well as coded comorbidity data. A full description of these methods, including the LLM prompt and comparison to coded diagnoses, can be found in the online supplemental Methods.

### Statistical analysis

Statistical analyses were conducted in R (V.4.3.1).

Patients with IBDU were, in keeping with revised Porto and Paris classifications, pragmatically allocated a binary label—that is, ‘IBDU-U, Crohn’s-type’ as Crohn’s disease and ‘IBD-U, colitis-type’ as ulcerative colitis. IBDU without suggestion of subtype was categorised as UC. This approach also supported statistical robustness by avoiding sparsely populated subgroups, allowing meaningful downstream comparison between two categories.^13 14^ Contemporary work positions PSC, AIH and overlap syndrome as a continuum of an overlapping, shared disease process which, as individually rare outcomes, were pragmatically grouped (IBDALD).^15^ Wilcoxon rank-sum testing was performed to compare biomarker values at IBD diagnosis (±6 months) between AIH/AIH-PSC overlap and PSC cases, ensuring no single subgroup unduly influenced downstream analyses. Non-hepatic causes of liver function test abnormalities (eg, muscle injury, systemic inflammation) were not specifically queried or adjusted for, as these are common, often transient and expected to be non-differentially distributed across IBDALD and non-IBDALD groups.^16^

To compare IBD-associated liver cases and controls, we performed an unpaired comparison of patient-level medians within ±6 months of IBD diagnosis. Participants were only included if they had at least one biomarker result available in the analysed timeframe and no data were imputed. Sensitivity analyses were undertaken using variable timeframes (0, 3, 6 and 12 months) around IBD diagnosis (online supplemental Tables 1 and 2). The ±6-month window was ultimately selected as it provided the optimal balance between clinical relevance and data completeness. Comparison was undertaken for continuous biomarkers and categorical labels from laboratory range flags (‘high’ vs ‘low’ and ‘normal’). A ‘low’ flag was interpreted as ‘normal’ for liver biomarkers and inflammatory markers, except for albumin where ‘low’ was considered ‘abnormal’. While ‘low’ biomarker values may represent abnormal physiology, this was felt unlikely to reflect hepatobiliary disease.^17^ Wilcoxon rank-sum tests were undertaken for continuous variables and Fisher’s exact tests for categorical variables.

A matched case-control design, using patient-level biomarker medians within ±6 months of IBD diagnosis, was implemented using nearest-neighbour 1:4 matching on age at diagnosis, gender and IBD subtype. To assess the robustness of matched case-control findings, sensitivity analyses were performed varying the matching ratio between IBD-ALD cases and non-IBD-ALD controls (1:2, 1:4 and 1:6) (online supplemental Table 3). Conditional logistic regression models were then fitted to evaluate associations between biomarker values and IBDALD case status. Models were run for individual biomarkers and in combination. Results were analysed as continuous variables and categorical indicators of abnormality based on laboratory range flags. Participants with a single ‘abnormal’ range flag over the period of interest were allocated to this group.

Exploratory visualisation of longitudinal biomarkers and their differences across groups was undertaken to determine overall distribution and temporal patterns. A density plot was generated to illustrate the overall distribution and frequency of biomarker testing across the full cohort over time. For biomarkers of interest (ALT, ALP, ESR, CRP, ALB and TB), LOESS (locally estimated scatterplot smoothing) curves were generated to examine the distribution of test values over time. For these plots, time was aligned such that month zero represented point of IBD-related liver disease or most recent follow-up in those unaffected. LOESS graphs were created using default package spans and improved by applying different span values to the IBDALD group, reducing overfitting in the smaller sample. Span value adjustment to 0.95 produced smoother trend lines in this group.

To test the longitudinal differences of biomarkers across groups, we modelled them via linear mixed-effects models (LMM) to assess their significance. LMMs included fixed effects for time (months before diagnosis or last follow-up), group (IBDALD vs non-IBDALD) and their interaction, with a random intercept, accounting for individual level variance. Random slopes were considered but excluded due to group imbalance, which risked model overfitting and instability. We began with an intercept-only model including time, group (IBD-associated liver disease vs non-associated) and their interaction. Models were then extended iteratively with covariates; gender, presence of all-cause liver comorbidity, IBD subtype and all combinations. The final full model for each log-transformed biomarker model included all covariates. Individuals with fewer than two longitudinal biomarker results were not analysed.

Model performance was evaluated using Akaike information criterion (AIC), Bayesian information criterion (BIC) and log-likelihood. Marginal and conditional R² values were calculated. Likelihood ratio tests were used to compare models against the base model. To overcome bias, residual analysis was undertaken. Assumptions were assessed for both base and full models, including normality of residuals (Shapiro-Wilk), skewness and kurtosis. Homoscedasticity was tested using Breusch-Pagan tests on linearised versions of the models. Q-Q plots and residual versus fitted value plots were generated for visual inspection.

All statistical tests were two-tailed; significance was defined as p<0.05. Missing biomarker data were not imputed. A STROBE (Strengthening the Reporting of Observational Studies in Epidemiology) checklist was completed and is available online in the online supplemental file 3 (STROBE checklist).

### Ethics statement

The ‘Genetics of IBD’ Study was approved by Southampton and South-West Hampshire Research Ethics Committee (09 /H0504/125). Written informed consent was provided by patients and/or their parents/legal guardians.

## Results

### Demographics

Data were extracted for the 1571 participants enrolled in the ‘Genetics of IBD’ study. The total number of females was 758 (48.2%). Nine hundred and eighty-six individuals had CD (62.8%) and the median age of IBD diagnosis was 18.0 years (range 1.3–88.8). Median total follow-up duration was 11.5 years (range 0.5–59.5). Thirty-five individuals had IBDALD (27 PSC, 2 AIH, 6 PSC/AIH overlap), of which 6 (4 PSC, 1 AIH, 1 PSC/AIH overlap) were diagnosed with IBDALD before or up to 3 months after IBD diagnosis and were excluded from analysis. Results of LLM methods utilised to identify these cases can be found in Online supplemental file 2. A summary of cohort demographics can be visualised in table 1. Comparison of biomarker values at IBD diagnosis (±6 months) between AIH/AIH-PSC overlap and PSC cases revealed no significant differences across biomarkers (online supplemental table 4), indicating that no single subgroup disproportionately influenced downstream analyses.

**Table 1:** A summary of the characteristics of the studied total cohort

Demographic variable	Total (percentage, %) or median, decimalised years (range)
Total participants	1571
Male	813 (51.8%)
Female	758 (48.2%)
Crohn’s disease	986 (62.8%)
Ulcerative colitis	585 (37.2%)
Diagnosed <17 years	606 (38.6%)
Diagnosed ≥17 years	965 (61.4%)
Age at IBD diagnosis (years), median (range)	18 (1.3–88.8)
Total follow-up duration	11.5 (0.5–59.5)
Participants with IBD-associated liver disease	35 (27 PSC, 2 AIH, 6 PSC/AIH overlap)
Time from IBD to associated liver diagnosis	4.5 (0.4–39.1)
Diagnosed with IBD <17 years	22 (62.9%)
Age at liver disease diagnosis (years), median (range)	18.3 (7–65.8)
Diagnosed with liver disease before IBD diagnosis	6 (4 PSC, 1 AIH, 1 PSC/AIH overlap)

AIH, autoimmune hepatitis; IBD, inflammatory bowel disease; PSC, primary sclerosing cholangitis.

### Clinical data

A total of 335 605 biomarker datapoints were included, a breakdown of test availability can be visualised in online supplemental table 4, and a density plot, demonstrating the distribution of these biomarkers across total cohort longitudinal disease course, can be viewed in online supplemental figure 1.

### Comparison of continuous biomarkers at IBD diagnosis

Sensitivity analyses comparing alternative diagnostic windows (±0, ±3, ±6 and ±12 months) yielded consistent findings (online supplemental Tables 1 and 2). Significant differences in several median biomarker levels were observed between IBDALD cases and non-IBDALD at IBD diagnosis ±6 months. A summary of these data can be visualised in table 2, including the number of cases for whom data were available at the selected timepoints. Median ALT for the IBDALD cohort was 30 IU/L (range 11–190 IU/L) and 16 IU/L (range 4.5–350 IU/L) for the non-liver disease group (p=4.52e-06). Median ALP also demonstrated a significant difference between groups; 182 IU/L (range 75–554 IU/L) in IBDALD and 102.75 IU/L (range 25–671.5 IU/L) for non-LD (p=1.83e-05). Median ESR was significantly different (p=0.026), 23.5 mm/hour (range 2–66.5 mm/hour) for IBDALD and 13 mm/hour (range 1–98 mm/hour) for non-IBDALD. GGT (p=8.73e-04) and AST also demonstrated significant differences (p=0.0228), although fewer results were available for the total cohort over this timeframe (n=75 for GGT; n=72 AST). Albumin (IBDALD 38 g/L (range 26–48 g/L); non-IBDALD 37 g/L (range:12–50); p=0.11) and TB (IBDALD 7 umol/L (range 4–20 umol/L); non-IBDALD 7 umol/L (range 2–33 umol/L); p=0.34) did not differ significantly between groups.

**Table 2:** Results of unpaired Wilcoxon rank-sum testing of median biomarker levels at IBD diagnosis ±6 months between IBD-associated liver disease cases and non-cases.

Biomarker	n (IBD-associated liver disease)	n (controls)	Median (range) – Cases	Median (range) – Controls	P value
ALT	19	1119	30 (11–190)	16 (4.5–350)	**4.16e-06**
ALP	19	1020	182 (75–554)	102.75 (25–671.5)	**1.62e-05**
GGT	5	69	89 (70–183)	13 (4–289)	**0.0009**
AST	4	65	34.25 (29–96.5)	22 (10–194)	**0.0186**
ESR	16	859	23.5 (2–66.5)	13 (1–98)	**0.0250**
ALB	19	1054	38 (26–48)	37 (12–50)	0.1140
TB	19	1087	7 (4–20)	7 (2–33)	0.3292
CRP	19	1100	2 (1–28)	5 (1–202)	**0.0310**

P values in bold demonstrate statistical significance <0.05.

ALB, albumin; ALP, alkaline phosphatase; AST, aspartate aminotransferase; CRP, C-reactive protein; ESR, erythrocyte sedimentation rate; GGT, gamma-glutamyl transferase; IBD, inflammatory bowel disease; IBD, inflammatory bowel disease; TB, total bilirubin.

### Comparison of normal versus abnormal biomarkers at IBD diagnosis

Significant differences were observed in the proportion of patients with abnormal biomarker values between IBDALD and non-IBDALD (online supplemental Table 6). ALT was abnormal in 63.2% of IBDALD compared with 29.6% of non-IBDALD (p=0.0037). ALP demonstrated a significant difference, with 47.4% of cases having an abnormal result vs 14.5% of non-IBDALD (p=0.0003). ESR also differed significantly between groups (p=0.0037), with abnormalities in 87.5% of IBDALD compared with 61.1% of non-IBDALD. GGT abnormalities were found in five IBDALD cases with results available compared with 14.3% of controls (p=0.0002); however, sample size was limited (n=75 total results). CRP was abnormal more frequently in non-IBDLD (65.1%) than IBDALD (36.8%) with a significant inverse relationship (p=0.021). No significant differences were observed for AST (p=0.17), ALB (p=0.24) or TB (p=1.00).

### Adjusted comparison of biomarkers across groups via conditional logistic regression

Liver comorbidities, such as steatosis and chronic liver disease, were rare and primarily observed in non-cases. Due to minimal prevalence of liver comorbidities, these were not included in matching. A table summarising these comorbidities at the time of or prior to IBD diagnosis can be visualised in the online supplemental table 7.

**Figure 1:**
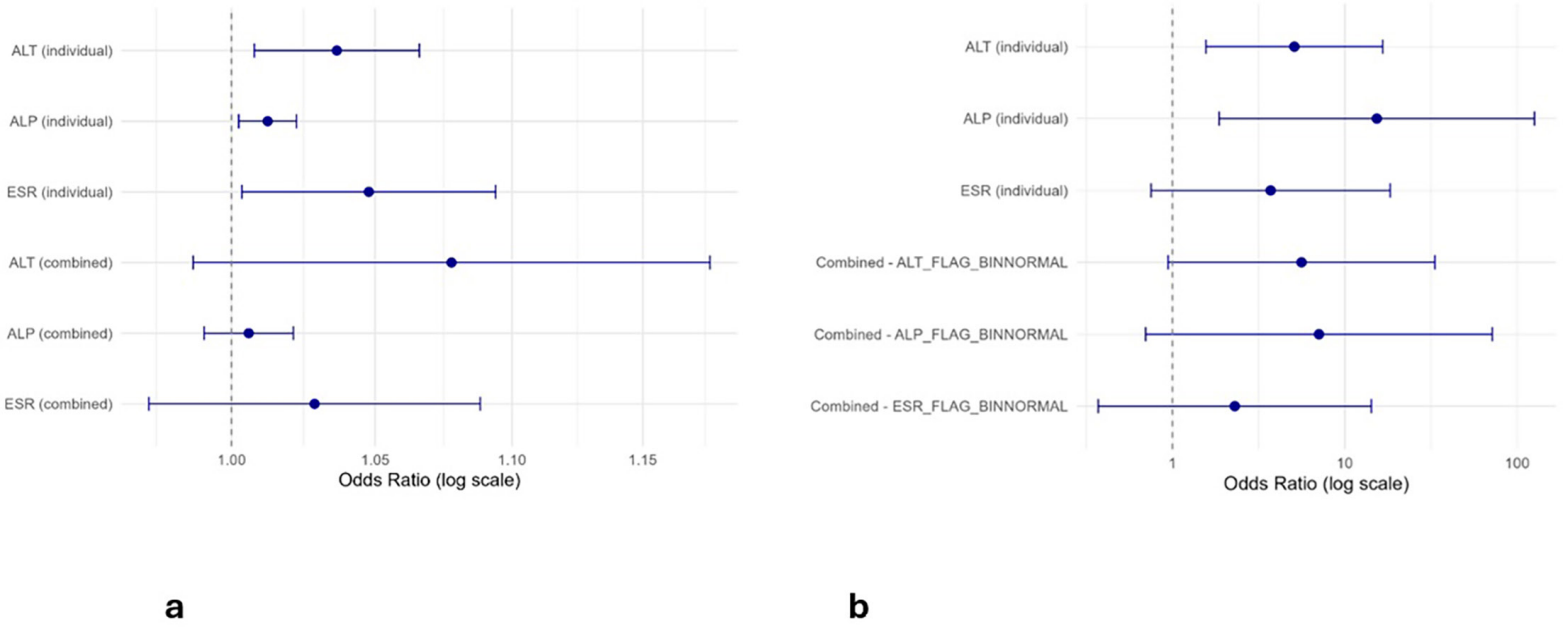
Graphs demonstrating the results of case-control matched model logistic regression using median biomarkers (a) and case-control matched model logistic regression utilising biomarker range flags (b). ALP, alkaline phosphatase; ALT, alanine aminotransferase; ESR, erythrocyte sedimentation rate.

In matched case-control models, ALT, ALP and ESR were all significantly associated with IBDALD when modelled individually and as continuous variables. Median ALT levels showed a significant association, with an OR of 1.04 (95% CI 1.01 to 1.07, p=0.012), suggesting that for each unit increase in ALT, the odds of IBDALD increased by 4%. Similarly, ALP was associated with IBDALD with an OR of 1.01 (95% CI 1.00 to 1.02, p=0.014), as was ESR (OR=1.05, 95% CI 1.00 to 1.09, p=0.034). In the combined model including ALT, ALP and ESR, none of the biomarkers remained statistically significant: ALT (OR=1.08, 95% CI 0.99 to 1.18, p=0.095), ALP (OR=1.01, 95% CI 0.99 to 1.02, p=0.451) and ESR (OR=1.03, 95% CI 0.97 to 1.09, p=0.326). The summary of these models can be visualised in online supplemental table 7 and figure 1.

When biomarker abnormalities were treated as binary variables using range flags, the association with IBDALD was more pronounced. Individuals with at least one high ALT result had over fivefold increased odds of IBDALD (OR=5.10, 95% CI 1.57 to 16.59, p=0.0068). Similarly, ALP abnormalities were strongly associated (OR=15.33, 95% CI 1.87 to 125.77, p=0.011). ESR did not reach statistical significance (OR=3.71, 95% CI 0.75 to 18.32, p=0.107). In the combined model including all three biomarkers, ALT (OR=5.61, 95% CI 0.94 to 33.32, p=0.058), ALP (OR=7.08, 95% CI 0.70 to 71.63, p=0.097) and ESR (OR=2.30, 95% CI 0.37 to 14.25, p=0.371) did not reach significance. The summary of these models can be visualised in online supplemental table 9 and figure 1**.**

ORs and statistical significance remained consistent across matching ratios, demonstrating stability of association strength for ALT, ALP and ESR biomarkers (online supplemental table 3). Increasing the matching ratio from 1:2 to 1:6 did not materially change effect estimates, indicating diminishing gain in precision beyond a 1:4 ratio.

### Exploration of the longitudinal course of biomarkers between groups

**Figure 2:**
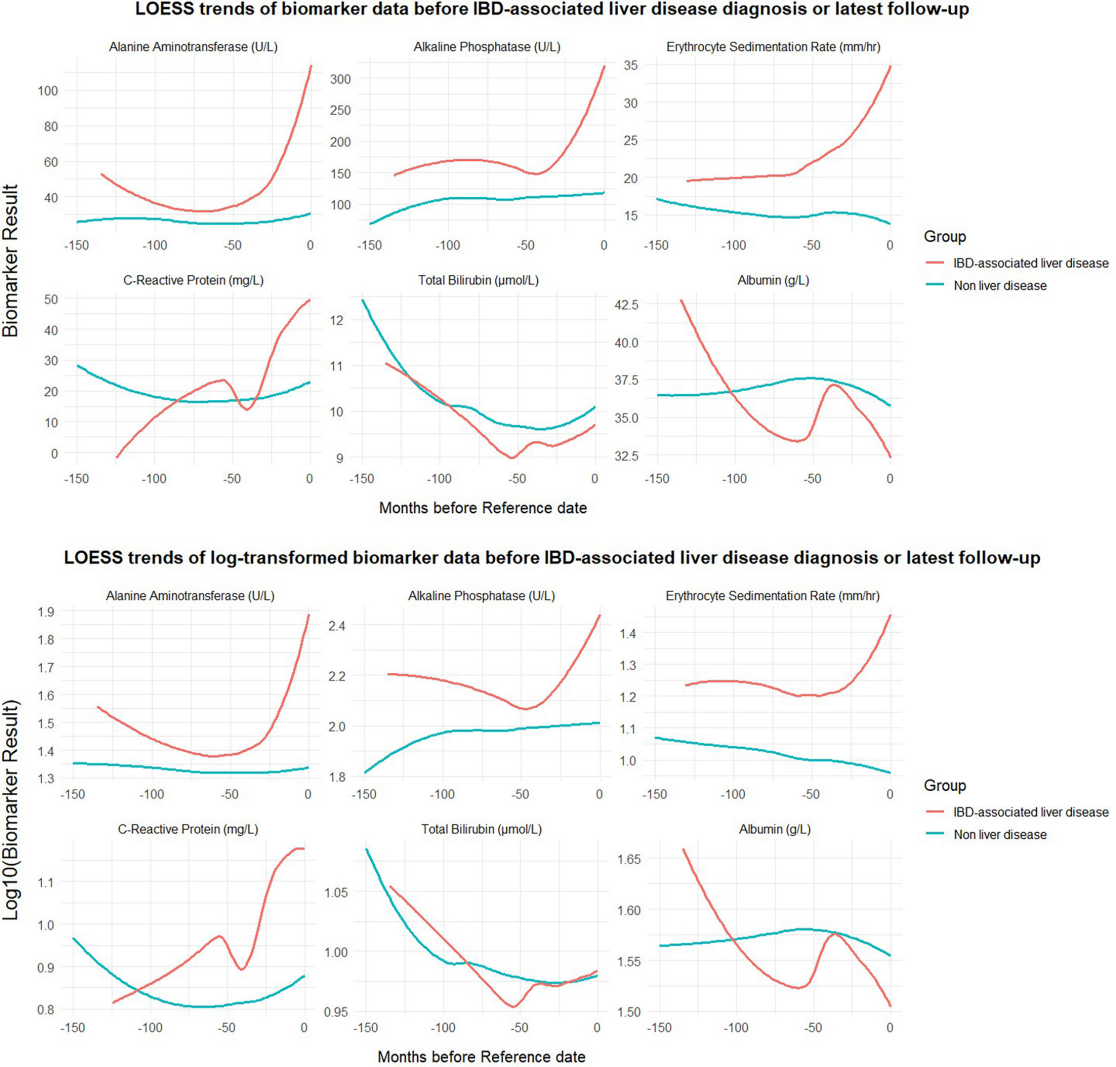
LOESS curves demonstrating longitudinal trends in raw biomarker and log10 adjusted results between IBD-associated liver disease and non-liver disease groups. IBD, inflammatory bowel disease; LOESS, locally estimated scatterplot smoothing.

LOESS plots suggest distinct longitudinal patterns of ALT, ALP and ESR between the two groups, indicating increasing values prior to diagnosis (see the red curves prior to reference time 0 in figure 2). CRP, ALB and bilirubin demonstrated overlapping trends.

### Comparison of longitudinal biomarker course

#### Alanine aminotransferase

Longitudinal ALT (log₁₀-transformed) was compared via LMM modelling where participant ALT profiles were aligned to the time of IBDALD diagnosis (cases, IBDALD=27) or last follow-up (non-IBDALD, n=1521). At this timepoint, patients with IBDALD had significantly higher ALT levels than non-IBDALD (*β*=–0.357, t=–11.36, p=1.02×10⁻²⁸). ALT values increased significantly over time in the IBDALD group (*β*=0.000354, t=2.72, p=0.0066), whereas they declined in the non-liver disease group (interaction term *β*=–0.000410, t=–3.12, p=0.0018), indicating diverging trajectories.

The base model was expanded by adding covariates into the model sequentially. Sequential covariate adjustment showed that both gender (*β*=0.075, p=7.30×10⁻¹⁸) and other liver comorbidity (*β*=0.131, p=5.52×10⁻³²) improved model performance (likelihood ratio p<2.2×10⁻¹⁶), while IBD subtype (CD) contributed minimally (*β*=–0.025, p=0.0063). The final model included group, time, gender, subtype and liver pathology, achieving the best model fit (AIC=–11057; marginal R²=0.095; conditional R²=0.412). Finally, the assumptions were checked via analysis of residuals. Residuals were approximately normally distributed (Shapiro-Wilk W=0.9252, p=1.13×10⁻⁴³); however, heteroscedasticity was detected (Breusch-Pagan test: BP=1306.19, p<2.2 × 10⁻¹⁶). Skewness (1.05) and kurtosis (4.89) were consistent with mild right tail-heaviness.

#### Alkaline phosphatase

Using the same approach, log₁₀(ALP) was modelled in 1468 patients (IBDALD=27, non-LD=1441). At the reference timepoint (0 months), the IBDALD group exhibited significantly higher ALP levels than controls (*β*=–0.235, t=–7.10, p=1.92×10⁻¹²). ALP values rose significantly over time in the IBDALD group (*β*=0.00037, t=4.24, p=2.25 × 10⁻⁵), with a steeper increase than in controls, reflected by a significant interaction term (*β*=–0.00086, t=–9.84, p=7.71×10⁻²³). These changes reflect a diverging trajectory in ALP over time, with faster elevation in affected individuals in the lead-up to diagnosis.

Residuals were approximately normally distributed (Shapiro-Wilk W=0.9621, p=2.75 × 10⁻³⁴). Heteroscedasticity was detected (Breusch-Pagan p<2.2×10⁻¹⁶). Skewness and kurtosis were modest (1.06 and 4.89). Covariate testing showed that gender (*β*=0.088, p=1.9×10⁻¹⁹) and other liver pathology (*β*=–0.025, p=0.041) significantly improved model fit. IBD subtype (CD) did not (*β*=0.0009, p=0.93). The final full model, which included group, time, gender, CD and liver pathology, demonstrated the strongest fit (AIC=–68241; marginal R²=0.086; conditional R²=0.677).

#### Erythrocyte sedimentation rate

A total of 1351 patients (IBDALD=24, non-IBDALD=1327) had ESR data available for modelling. At timepoint zero, patients in the IBDALD group had significantly higher ESR values (*β*=–0.247, t=–4.47, p=8.6×10⁻⁶). There was no evidence of a linear association with time in either group (*β*=0.00018, t=0.40, p=0.69), and the interaction term was non-significant (*β*=0.00057, t=1.29, p=0.20), indicating parallel longitudinal trajectories.

Residuals were approximately normally distributed (Shapiro-W=0.993) and homoscedastic (Breusch-Pagan p=0.74). The final full model (AIC=9316, marginal R²=0.059, conditional R²=0.480) included group, time, gender, CD diagnosis and other liver pathology. Within this model, ESR was significantly lower in patients without liver disease (*β*=–0.297, t=–5.49, p=4.6×10⁻⁸), while male gender was associated with lower ESR (*β*=–0.115, t=–7.38, p=2.9×10⁻¹³). CD diagnosis and presence of other liver pathology were each associated with modestly increased ESR (CD: *β*=0.055, t=3.36, p=0.00079; Other liver pathology: *β*=0.079, t=4.13, p=3.9×10⁻⁵). The time-by-group interaction remained non-significant (*β*=0.00052, t=1.16, p=0.24). A summary of model performance metrics and fixed-effect estimates for base and fully adjusted linear mixed-effects models for ALT, ALP and ESR can be visualised in table 3.

**Table 3:** Summary of model performance metrics and fixed-effect estimates for base and fully adjusted linear mixed-effects models assessing longitudinal trends in log-10-transformed ALT, ALP and ESR

Parameter	ESR base	ESR full	ALP base	ALP full	ALT base	ALT full
AIC	9376.46	9316.21	–68 179.8	–68 241.0	–10 890.0	–11 057.2
BIC	9423.26	9386.41	–68 125.6	–68 159.8	–10 835.5	–10 975.4
log-likelihood	–4682.23	–4649.10	34 095.89	34 129.51	5451.02	5537.62
Marginal R² (fixed)	0.0157	0.0588	0.0410	0.0860	0.0352	0.0949
Conditional R² (full)	0.4752	0.4801	0.6738	0.6769	0.4069	0.4125
Likelihood ratio p value	–	2.19×10⁻¹⁸	–	3.28×10⁻¹⁹	–	3.15×10⁻⁴²
Intercept (β)	1.235	1.291	2.268	2.203	1.660	1.615
Time (months) (β)	0.00018	0.00020	0.00037	0.00036	0.00035	0.00034
Group: IBDALD vs non-LD (β)	–0.247	–0.297	–0.235	–0.211	–0.357	–0.361
Time×group (β)	0.00057	0.00052	–0.00086	–0.00086	–0.00041	–0.00040
Gender: male (β)	–0.115	–0.115	0.088	0.088	0.075	0.075
CD vs UC (β)	0.055	0.055	0.0009	0.0009	–0.025	–0.025
Other liver pathology (β)	0.079	0.079	–0.025	–0.025	0.131	0.131

AIC, Akaike information criterion; ALP, alkaline phosphatase; ALT, alanine aminotransferase; BIC, Bayesian infiormation criterion; CD, Crohn’s disease; ESR, erythrocyte sedimentation rate; IBDALD, inflammatory bowel disease-associated liver disease; LD, liver disease; UC, ulcerative colitis.

Base models included fixed effects for time (months before IBDALD diagnosis or latest follow-up), group (IBDALD vs non-liver disease) and their interaction, with a random intercept for individual. Full models include adjustment for gender (male vs female), IBD subtype (CD vs UC) and other liver pathology (excluding IBDALD). Intercept (β) refers to the estimated biomarker value at timepoint zero for the reference group. Time (β) denotes the monthly rate of biomarker change in non-liver disease controls; interaction terms reflect group differences in trajectory. Marginal R² represents the variance explained by fixed effects; conditional R² includes both fixed and random effects. Likelihood ratio test p values compare full models against respective base models.

## Discussion

In this study, we evaluated routinely collected liver and inflammatory biomarkers for their association with the development of IBDALD in a well-characterised IBD cohort (n=1571). IBDALD was identified in 35 individuals, predominantly with PSC. Across unpaired analyses, matched case-control comparisons and longitudinal modelling, significant elevations in ALT, ALP and ESR were observed in individuals who developed IBDALD, including at the time of IBD diagnosis. These biomarkers remained associated with IBDALD after adjustment for potential confounders. Longitudinal trajectories of ALT and ALP also diverged significantly in the years preceding IBDALD diagnosis, highlighting potential as early predictive biomarkers of liver involvement in IBD.

Elevated ALT, ALP and ESR values within 6 months of IBD diagnosis were associated with increased odds of IBDALD. Likelihood of developing IBDALD was higher when individuals had a single ‘abnormal’ ALP, ALT or ESR within this timeframe. ORs were highest for ALP, supporting its known role as a marker of cholestasis. Matched case-control methods were carried out to ensure that comparisons of these biomarker levels were not biased by the covariates of age, gender, IBD subtype and other liver pathology. When biomarkers were modelled jointly, individual biomarker significance decreased, indicating potential collinearity or shared variance between markers.

Longitudinal analysis provided further insight into biomarker dynamics. Distinct temporal trajectories were observed for ALT, ESR and ALP in LOESS plots. ESR was elevated at the time of IBD diagnosis in patients who later developed IBDALD, but it exhibited parallel longitudinal trajectories across groups, suggesting it may function more as a non-specific marker of systemic inflammation reflecting underlying IBD activity or other extraintestinal manifestations—rather than a dynamic predictor of liver disease evolution. In contrast, both ALT and ALP demonstrated accelerated—although small—rises prior to IBDALD diagnosis, supporting a potential role in preclinical detection. All models fit improved with the inclusion of covariates gender and liver comorbidity, reinforcing the importance of contextualising biomarker trends within known patient-specific factors. ALT and ALP models exhibited heteroscedasticity, a common feature in large-scale biomarker datasets, likely reflecting underlying individual biological variability.

Importantly, while transient abnormalities in liver function tests such as ALT and ALP are common in IBD—often reflecting inflammation, medication effects or non-specific hepatobiliary changes—IBDALD remains rare. This disparity highlights the vital clinical challenge of distinguishing benign derangement from early signals of evolving pathology. Although effect sizes were modest, our findings suggest that risk stratification is feasible using routinely collected biomarker data. The observed divergence in longitudinal ALT and ALP trends may help distinguish consistent biomarker changes associated with disease from transient, non-specific fluctuations. This represents an immediately translatable approach to identify individuals at elevated risk who may benefit from enhanced surveillance, such as earlier or more frequent hepatobiliary imaging and targeted clinical follow-up. Identifying patterns or trajectories that differentiate high-risk individuals is therefore of critical relevance and underscores the utility of predictive modelling applied to real-world longitudinal data.

Categorising biomarker data into binary range-flag values (eg, ‘high’ vs ‘normal’) enhances clinical interpretability by aligning with conventional diagnostic thresholds. This simplification can aid communication and model deployment in clinical settings. However, binarisation reduces sensitivity to detect subtle within-range variation and may exaggerate effect sizes due to threshold-based grouping. As such, binary and continuous models should be interpreted in parallel to balance clinical utility with statistical robustness. Furthermore, biomarkers such as AST and GGT, despite showing significant group differences, were available for only a minority of participants and are typically ordered in response to clinical suspicion, introducing selection bias.^3^

Existing literature on IBDALDs has primarily focused on descriptive cohort studies and genetic risk profiling, with limited investigation into biomarker trends.^6 18^ While clustering patients with IBD based on biomarker trajectories to predict disease course has been described, this approach has yet to achieve clinical utility.^7 19^ There have, however, been calls for longitudinal assessments of IBD activity, particularly those emphasising clinically relevant outcomes, including extraintestinal manifestations, and for the use of clinical data to support prognostication and personalised care.^19^ Notably, there is a paucity of studies applying advanced data-driven approaches, specifically to IBDALD, representing a critical gap in the literature. Our study contributes to this direction by demonstrating potential biomarker signals.

As demonstrated both in the literature and within our cohort, the prevalence of IBDALD remains low, even in large, real-world IBD populations. While the examined cohort is large, a preponderance of younger individuals (median age of diagnosis 18.0 years) with variable follow-up duration may limit capture of later-onset liver complications. It is therefore possible that some non-IBDALD individuals may go on to develop liver disease over time, underscoring the importance of longitudinal validation and extended follow-up in future studies. Future research should focus on expanding cohort size through prospective patient recruitment and collaboration across cohorts to improve detection and validation of these predictive signals. Larger cohorts will be required to enable age-specific and disease-specific subgroup analyses, which were not statistically meaningful within the current dataset. Integration of genomic and multiomic data alongside biomarker trends could enhance the resolution of potential risk stratification models.^7 20^

Strengths of this study include the integration of highly dimensional, routinely collected clinical data with LLM-assisted phenotyping, and the use of multiple complementary modelling approaches to triangulate findings. A potential limitation is the relatively small number of IBDALD cases, which constrained subgroup analyses and could limit generalisability. However, data are from a real-world cohort with PSC prevalence rates reflective of other observed cohorts where, in UC alone, rates of 3–14% have been reported.^21^ Additionally, our cohort demonstrates a preponderance for CD (62.8%). Oversampling or artificially increasing case representation was avoided, as these techniques would limit the real-world applicability of the predictive models. The use of real-world data inherently involves some missingness, leading to exclusion of a small number of patients from specific analyses; however, data were not imputed to preserve the clinical validity of the dataset.

We aimed to reduce the influence of covariates such as gender, disease subtype and liver comorbidity; however, we acknowledge that IBD therapies can impact liver biomarkers, data for which were not available in this dataset. Moreover, prescription data for relevant hepatoprotective agents, including ursodeoxycholic acid, were not available in this study. However, use is assumed to be comparable across IBD-ALD and non-IBD-ALD groups prior to liver disease diagnosis and therefore unlikely to have introduced systematic bias into model estimates. Future work could incorporate treatment data, although the typically transient elevations in liver function tests associated with certain medications are unlikely to significantly influence long-term trends. Importantly, the associations demonstrated were evident despite the low number of IBDALD events and even prior to adjustment for liver comorbidities, highlighting the observed signal and supporting the potential role of these routine biomarkers as early indicators warranting closer monitoring in this patient group.

## Conclusion

Our findings show that routinely collected biomarkers, particularly ALT and ALP, exhibit early predictive signals for IBDALD, with distinct longitudinal trends preceding diagnosis. Although effect sizes are modest, these measurable and clinically relevant changes provide a practical means of risk stratification in real-world IBD populations. Careful monitoring of liver biomarkers at diagnosis and confirmation of their normalisation during follow-up may help to identify individuals at increased risk, prompting earlier hepatobiliary assessment and reducing diagnostic delay. These results support the utility of large-scale, routinely collected clinical data to enhance early detection and guide preventive strategies for extraintestinal complications in IBD. Replication of these findings in larger, more diverse and international cohorts will be essential to confirm generalisability and strengthen translational relevance.

## Supplementary material

10.1136/bmjgast-2025-002028 online supplemental file 1

10.1136/bmjgast-2025-002028 online supplemental file 2

10.1136/bmjgast-2025-002028 online supplemental file 3

## Data Availability

No data are available.
