# Supplementary material for: Risk stratification of IBD-associated liver disease using routinely collected biomarkers from a large-scale real-world dataset
Source: BMJ Open Gastroenterol. 2025 Nov 13;12(1):e002028. doi: 10.1136/bmjgast-2025-002028 (PMC12625833; doi:10.1136/bmjgast-2025-002028)
Supplement: online supplemental file 1 [file bmjgast-12-1-s001.docx]

**SUPPLEMENTARY FIGURES AND TABLES**


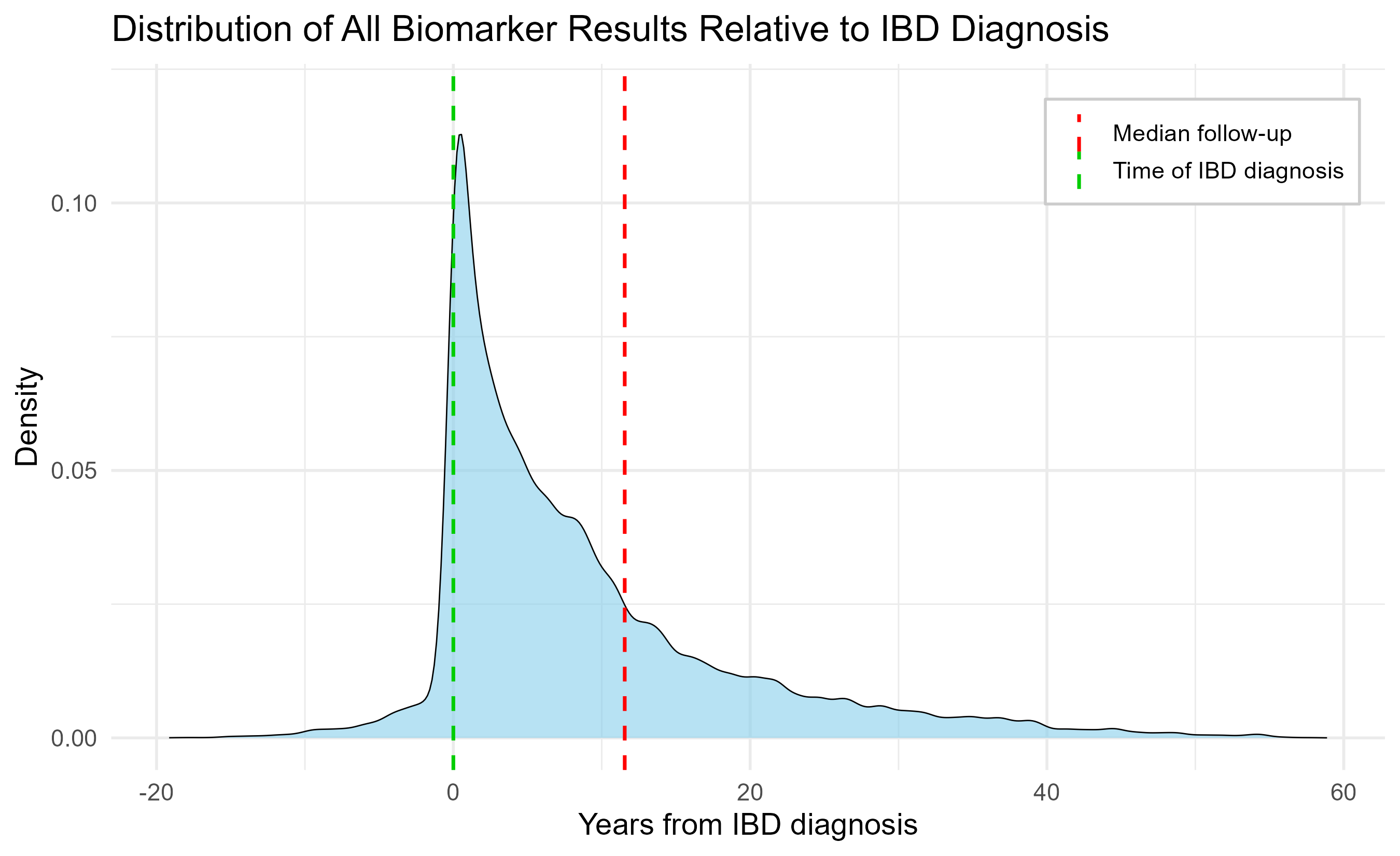


***SUPPLEMENTARY FIGURE 1: A density plot demonstrating the distribution of biomarkers sampled across the total cohort (n=1,571) in relation to IBD diagnosis.***

| Test | Time  months | n (IBD-ALD) | n (non-IBDALD) | Median (Range) – Cases | Median (Range) – non-IBDALD | p-value |
| --- | --- | --- | --- | --- | --- | --- |
| ALB | ±0m | 14 | 661 | 36.5 (24–41) | 33 (1–48) | 0.110678 |
| ALB | ±3m | 18 | 994 | 37.5 (32–45) | 36 (5–50) | **0.044606** |
| ALB | ±6m | 19 | 1054 | 38 (26–48) | 37 (12–50) | 0.113589 |
| ALB | ±12m | 20 | 1096 | 38.25 (25–48) | 37.5 (12–49) | 0.199786 |
| ALP | ±0m | 14 | 640 | 280.5 (98–802) | 105 (24–1029) | **1.21E-06** |
| ALP | ±3m | 18 | 957 | 185.25 (99–554) | 101 (25–770) | **8.51E-07** |
| ALP | ±6m | 19 | 1020 | 182 (75–554) | 102.25 (25–671.5) | **1.62E-05** |
| ALP | ±12m | 20 | 1059 | 177 (67.5–573.5) | 103 (24.5–671.5) | **2.06E-05** |
| ALT | ±0m | 14 | 694 | 86.5 (11.5–276.5) | 15 (3–546.5) | **8.16E-07** |
| ALT | ±3m | 18 | 1051 | 40.25 (11–190) | 16 (4.5–442) | **2.17E-05** |
| ALT | ±6m | 19 | 1119 | 30 (11–190) | 16 (4.5–350) | **4.16E-06** |
| ALT | ±12m | 20 | 1162 | 37.25 (11–190) | 16 (5–350) | **1.37E-07** |
| AST | ±0m | 0 | 16 | NA | 22 (12–358) |  |
| AST | ±3m | 4 | 43 | 33.5 (26–108.5) | 22 (10–194) | **0.075931** |
| AST | ±6m | 4 | 65 | 34.25 (29–96.5) | 22 (10–194) | **0.018624** |
| AST | ±12m | 5 | 88 | 35.5 (29–96.5) | 22.5 (10–180) | **0.005293** |
| CRP | ±0m | 13 | 672 | 3 (1–92) | 12 (1–279) | 0.132899 |
| CRP | ±3m | 18 | 1029 | 2.25 (1–52) | 6.1 (1–199) | **0.049909** |
| CRP | ±6m | 19 | 1100 | 2 (1–28) | 5 (1–202) | **0.031005** |
| CRP | ±12m | 20 | 1148 | 2 (1–27) | 4.5 (1–202) | 0.088344 |
| ESR | ±0m | 10 | 377 | 38.25 (14–108) | 24 (1–118) | **0.016684** |
| ESR | ±3m | 14 | 765 | 25 (8–67) | 15 (1–118) | **0.02776** |
| ESR | ±6m | 16 | 859 | 23.5 (2–66.5) | 13 (1–98) | **0.025094** |
| ESR | ±12m | 17 | 959 | 23 (2–66.5) | 12 (1–136) | **0.008572** |
| GGT | ±0m | 2 | 20 | 79.5 (70–89) | 13 (5–49) | **0.025608** |
| GGT | ±3m | 5 | 47 | 74 (62–224) | 14 (3–289) | **0.002332** |
| GGT | ±6m | 5 | 69 | 89 (70–183) | 13 (4–289) | **0.000896** |
| GGT | ±12m | 5 | 86 | 89 (57–183) | 13 (4–289) | **0.000792** |
| TB | ±0m | 14 | 678 | 7.75 (2–13) | 6 (2–38) | 0.224084 |
| TB | ±3m | 18 | 1019 | 7 (4–16) | 7 (2–37.5) | 0.612707 |
| TB | ±6m | 19 | 1087 | 7 (4–20) | 7 (2–33) | 0.329198 |
| TB | ±12m | 20 | 1126 | 8 (4–23) | 7 (2–35) | 0.36656 |
| *SUPPLEMENTARY TABLE 1: Sensitivity analysis for results of unpaired Wilcoxon rank-sum testing of median biomarker levels at IBD diagnosis with variable timeframe (months) between IBD-associated liver disease cases and non-cases* | | | | | | |

| Test | Time  Months | n (IBD-ALD) | n (non-IBDALD) | Abnormal (%) Cases | Abnormal (%) Controls | p-value |
| --- | --- | --- | --- | --- | --- | --- |
| ALB | ±0m | 14 | 661 | 42.9% | 51.8% | 0.594039 |
| ALB | ±3m | 18 | 994 | 33.3% | 46.4% | 0.34279 |
| ALB | ±6m | 19 | 1054 | 31.6% | 47.7% | 0.173946 |
| ALB | ±12m | 20 | 1096 | 30% | 50.2% | 0.112112 |
| ALP | ±0m | 14 | 640 | 42.9% | 7.5% | **0.000432** |
| ALP | ±3m | 18 | 957 | 44.4% | 11.6% | **0.000596** |
| ALP | ±6m | 19 | 1020 | 47.4% | 14.2% | **0.000675** |
| ALP | ±12m | 20 | 1059 | 55% | 18.6% | **0.00035** |
| ALT | ±0m | 14 | 694 | 71.4% | 18.8% | **3.48E-05** |
| ALT | ±3m | 18 | 1051 | 66.7% | 25.8% | **0.000368** |
| ALT | ±6m | 19 | 1119 | 63.2% | 29.4% | **0.003673** |
| ALT | ±12m | 20 | 1162 | 75% | 34.7% | **0.000465** |
| AST | ±0m | 0 | 16 | NA | 12.5% | NA |
| AST | ±3m | 4 | 43 | 50% | 14% | 0.12896 |
| AST | ±6m | 4 | 65 | 50% | 15.4% | 0.136924 |
| AST | ±12m | 5 | 88 | 60% | 13.6% | **0.028397** |
| CRP | ±0m | 13 | 672 | 38.5% | 66.3% | **0.071306** |
| CRP | ±3m | 18 | 1029 | 38.9% | 63.3% | **0.046728** |
| CRP | ±6m | 19 | 1100 | 36.8% | 64.9% | **0.015056** |
| CRP | ±12m | 20 | 1148 | 40% | 69.6% | **0.006862** |
| ESR | ±0m | 10 | 377 | 100% | 69.8% | **0.037692** |
| ESR | ±3m | 14 | 765 | 85.7% | 60% | **0.056666** |
| ESR | ±6m | 16 | 859 | 87.5% | 61.5% | **0.037458** |
| ESR | ±12m | 17 | 959 | 88.2% | 63.1% | **0.039959** |
| GGT | ±0m | 2 | 20 | 100% | 5% | **0.012987** |
| GGT | ±3m | 5 | 47 | 100% | 17% | **0.000495** |
| GGT | ±6m | 5 | 69 | 100% | 13% | **0.000124** |
| GGT | ±12m | 5 | 86 | 100% | 15.1% | **0.000184** |
| TB | ±0m | 14 | 678 | 0% | 2.8% | 1 |
| TB | ±3m | 18 | 1019 | 5.6% | 5.6% | 1 |
| TB | ±6m | 19 | 1087 | 5.3% | 8.5% | 1 |
| TB | ±12m | 20 | 1126 | 15% | 12.4% | 0.729678 |
| *SUPPLEMENTARY TABLE 2: Sensitivity analysis for results of Fisher’s exact test reported for each biomarker by range flag at IBD diagnosis with variable timeframe (months) between IBD-associated liver disease cases and non-IBDALD* | | | | | | |

| label | odds_ratio | ci_lower | ci_upper | p | ratio | n_total | IBDALD | Non_IBDALD |
| --- | --- | --- | --- | --- | --- | --- | --- | --- |
| ALT (individual) (MEDIAN_RESULT_ALT) | 1.029218226 | 1.001024 | 1.058206636 | **0.042135648** | 2 | 71 | 19 | 52 |
| ALP (individual) (MEDIAN_RESULT_ALP) | 1.01740522 | 1.003852 | 1.031141073 | **0.011672097** | 2 | 64 | 19 | 45 |
| ESR (individual) (MEDIAN_RESULT_ESR) | 1.076145558 | 1.001738 | 1.156080071 | **0.044700296** | 2 | 55 | 16 | 39 |
| ALT (individual) (MEDIAN_RESULT_ALT) | 1.036426319 | 1.007747 | 1.065921416 | **0.012454232** | 4 | 111 | 19 | 92 |
| ALP (individual) (MEDIAN_RESULT_ALP) | 1.012339927 | 1.002452 | 1.022325162 | **0.014323741** | 4 | 102 | 19 | 83 |
| ESR (individual) (MEDIAN_RESULT_ESR) | 1.047757646 | 1.003545 | 1.093917921 | **0.033934721** | 4 | 87 | 16 | 71 |
| ALT (individual) (MEDIAN_RESULT_ALT) | 1.035078261 | 1.010818 | 1.059920975 | **0.004383873** | 6 | 155 | 19 | 136 |
| ALP (individual) (MEDIAN_RESULT_ALP) | 1.013047341 | 1.003608 | 1.022574978 | **0.006644956** | 6 | 138 | 19 | 119 |
| ESR (individual) (MEDIAN_RESULT_ESR) | 1.052379796 | 1.010675 | 1.095805031 | **0.013335616** | 6 | 121 | 16 | 105 |
| MEDIAN_RESULT_ALT (combined) | 1.07162227 | 0.963202 | 1.19224608 | 0.203708021 | 2 | 49 | 16 | 33 |
| MEDIAN_RESULT_ALP (combined) | 1.002451445 | 0.985876 | 1.019305438 | 0.773482751 | 2 | 49 | 16 | 33 |
| MEDIAN_RESULT_ESR (combined) | 1.046723148 | 0.970548 | 1.128877213 | 0.236209811 | 2 | 49 | 16 | 33 |
| MEDIAN_RESULT_ALT (combined) | 1.077634961 | 0.987022 | 1.176566161 | 0.095222193 | 4 | 79 | 16 | 63 |
| MEDIAN_RESULT_ALP (combined) | 1.005848338 | 0.990707 | 1.021221546 | 0.451153305 | 4 | 79 | 16 | 63 |
| MEDIAN_RESULT_ESR (combined) | 1.028618943 | 0.972305 | 1.088194982 | 0.32597188 | 4 | 79 | 16 | 63 |
| MEDIAN_RESULT_ALT (combined) | 1.049907111 | 0.995002 | 1.107842067 | 0.07554763 | 6 | 107 | 16 | 91 |
| MEDIAN_RESULT_ALP (combined) | 1.008348868 | 0.994553 | 1.022335664 | 0.236839747 | 6 | 107 | 16 | 91 |
| MEDIAN_RESULT_ESR (combined) | 1.040955566 | 0.978137 | 1.107808944 | 0.206267658 | 6 | 107 | 16 | 91 |
| *SUPPLEMENTARY TABLE 3: Sensitivity analysis for variable ratios of case-control matched models (1:2, 1:4, 1:6) utilising median biomarker results at IBD diagnosis ±6 months. Models generated using clogit in R. Wald tests used to derive p values (signif. <0.05). OR= Odds Ratio* | | | | | | | | |

| Biomarker | | n (AIH/AIH-PSC) | n (PSC) | Median (Range) – AIH/AIH-PSC | Median (Range) – PSC | p-value |
| --- | --- | --- | --- | --- | --- | --- |
| ALB | 6 | 13 | 39.5 (32–42) | 38 (26–48) | 0.7249 |  |
| ALP | 6 | 13 | 216.5 (117–341) | 166 (75–554) | 0.6295 |  |
| ALT | 6 | 13 | 35.5 (13–107) | 30 (11–190) | 0.7924 |  |
| AST | 2 | 2 | 67.25 (38–96.5) | 29.75 (29–30.5) | 0.2453 |  |
| CRP | 6 | 13 | 1 (1–6.5) | 4 (1–28) | 0.126 |  |
| ESR | 6 | 10 | 26 (10.5–49) | 22.5 (2–66.5) | 0.6255 |  |
| GGT | 4 | 1 | 81.5 (70–183) | 96 (96–96) | 0.7237 |  |
| TB | 6 | 13 | 7.5 (6–14.5) | 7 (4–20) | 0.691 |  |
| *SUPPLEMENTARY TABLE 4: Comparative analysis median biomarker levels for AIH and AIH/PSC overlap with PSC at IBD diagnosis ±6 months - unpaired Wilcoxon rank-sum testing* | | | | | |  |

| Biomarker | n (IBD- associated liver disease) | n (Controls) | Abnormal (%) Controls | Abnormal (%) Cases | *p*-value |
| --- | --- | --- | --- | --- | --- |
| ALB | 19 | 1054 | 47.7% | 31.6% | 0.1739 |
| ALP | 19 | 1020 | 14.2% | 47.4% | **0.0007** |
| ALT | 19 | 1119 | 29.4% | 63.2% | **0.0037** |
| AST | 4 | 65 | 15.4% | 50.0% | 0.1369 |
| CRP | 19 | 1100 | 64.9% | 36.8% | **0.0150** |
| ESR | 16 | 859 | 61.5% | 87.5% | **0.0368** |
| GGT | 5 | 69 | 13% | 100.0% | **0.0001** |
| TB | 19 | 1087 | 8.5% | 5.3% | 1.000 |
| *SUPPLEMENTARY TABLE 6: Summary of biomarker results by range flag at IBD diagnosis ±6 months between IBD-associated liver disease cases and non-IBDALD. Fisher’s exact test reported for each biomarker.* | | | | | |

| Biomarker | Results (n) | Laboratory Normal range | Units |
| --- | --- | --- | --- |
| ALT | 65,743 | 0-49 | U/L |
| ALB | 64,139 | 35-50 | g/L |
| TB | 63,800 | 0-20 | umol/L |
| ALP | 61,613 | 30-130 | U/L |
| CRP | 60,826 | 0-5 | mg/L |
| ESR | 18,030 | 1-30 | mm/h |
| GGT | 775 | 0-37 | U/L |
| AST | 679 | 0-49 | iu/L |
| TOTAL | 335,605 |  |  |
| *SUPPLEMENTARY TABLE 5: Availability of biomarkers across total longitudinal data set, normal laboratory range values (adult) and units; ALT, alanine aminotransferase; ALP, alkaline phosphatase; TB, total bilirubin, AST, aspartate aminotransferase; GGT, gamma-glutamyl transferase; CRP, C-reactive protein; ESR, erythrocyte sedimentation rate; ALB, albumin* | | | |

| Comorbidity | Non-IBDALD (n = 1536) | IBD-associated liver disease Cases (n= 35) |
| --- | --- | --- |
| Steatosis | 54 | 0 |
| Cholestasis | 1 | 0 |
| Chronic Liver Disease | 2 | 0 |
| Infection | 10 | 5 |
| Structural | 33 | 0 |
| Hepatomegaly | 2 | 0 |
| *SUPPLEMENTARY TABLE 7: Liver Comorbidity Distribution by Group, pathologies identified for each co-morbidity heading are visualised in SUPPLEMENTARY TABLE A* | | |

| label | term | OR | ci_lower | ci_upper | p | n_total | IBDALD | Non-IBDALD |  |
| --- | --- | --- | --- | --- | --- | --- | --- | --- | --- |
| ALT (individual) | MEDIAN_RESULT_ALT | 1.036426 | 1.007747 | 1.065921 | 0.012454 | 111 | 19 | 92 |  |
| ALP (individual) | MEDIAN_RESULT_ALP | 1.01234 | 1.002452 | 1.022325 | 0.014324 | 102 | 19 | 83 |  |
| ESR (individual) | MEDIAN_RESULT_ESR | 1.047758 | 1.003545 | 1.093918 | 0.033935 | 87 | 16 | 71 |  |
| ALT (combined) | MEDIAN_RESULT_ALT | 1.077635 | 0.987022 | 1.176566 | 0.095222 | 79 | 16 | 63 |  |
| ALP (combined) | MEDIAN_RESULT_ALP | 1.005848 | 0.990707 | 1.021222 | 0.451153 | 79 | 16 | 63 |  |
| ESR (combined) | MEDIAN_RESULT_ESR | 1.028619 | 0.972305 | 1.088195 | 0.325972 | 79 | 16 | 63 |  |
| SUPPLEMENTARY TABLE 8: A summary of case-control matched models utilising median biomarker results *at IBD diagnosis ±6 months. Models generated using* clogit in R. Wald tests used to derive p values (signif. <0.05). OR= Odds Ratio | | | | | | | | | |

| Model | term | odds_ratio | ci_lower | ci_upper | p | n_total | n_cases | n_controls |
| --- | --- | --- | --- | --- | --- | --- | --- | --- |
| ALT (range flag only) | ALT_FLAG_BINNORMAL | 5.097091 | 1.565936 | 16.59093 | 0.006835 | 111 | 19 | 92 |
| ALP (range flag only) | ALP_FLAG_BINNORMAL | 15.32632 | 1.867642 | 125.7715 | 0.011034 | 102 | 19 | 83 |
| ESR (range flag only) | ESR_FLAG_BINNORMAL | 3.713329 | 0.75272 | 18.31865 | 0.107154 | 87 | 16 | 71 |
| Combined (range flags only) | ALT_FLAG_BINNORMAL | 5.609055 | 0.944306 | 33.31705 | 0.057838 | 79 | 16 | 63 |
| Combined (range flags only) | ALP_FLAG_BINNORMAL | 7.077403 | 0.699314 | 71.62679 | 0.097499 | 79 | 16 | 63 |
| Combined (range flags only) | ESR_FLAG_BINNORMAL | 2.300739 | 0.37137 | 14.25371 | 0.370549 | 79 | 16 | 63 |
| *SUPPLEMENTARY TABLE 9: A summary of conditional logistic regression models utilising biomarker range flag results at IBD diagnosis ±6 months. Models generated using clogit in R. Wald tests used to derive p values (signif. <0.05)* | | | | | | | | |
