# Supplementary material for: Risk stratification of IBD-associated liver disease using routinely collected biomarkers from a large-scale real-world dataset
Source: BMJ Open Gastroenterol. 2025 Nov 13;12(1):e002028. doi: 10.1136/bmjgast-2025-002028 (PMC12625833; doi:10.1136/bmjgast-2025-002028)
Supplement: online supplemental file 2 [file bmjgast-12-1-s002.docx]

**SUPPLEMENTARY METHODS: LARGE-LANGUAGE MODEL STRUCTURING OF CLINICAL REPORTS**

UHS unstructured clinical reports for the 1,571 in the total cohort have been anonymised using locally developed and deployed redacting software, *Pteredactyl* [1]. Records comprised of 32,041 files, including multi-modal imaging (n= 24,860; 77.59%) and histology (n=7,181; 22.41%) reports. These reports are securely and anonymously stored within the secure University of Southampton (UoS) high-performance computing cluster (HPC) IRIDIS X.

A local iteration of Llama 3.1 70B (llama3.1:8b-instruct-fp16), a large-language model (LLM), was utilised to query and structure these data. This model was downloaded using Ollama v0.3.12. The model was run on the IRIDIS X HPC using Dual NV-Linked A100 nodes (2x A100 per node, NV-linked, 80 GB of VRAM per GPU). A prompt was iteratively devised to extract and structure instances of specified IBD-associated liver pathology. This instruction extracted the instance of relevant liver findings from any part of the free-text reports:

**prompt = (**

**"Read the following medical report and perform the following tasks: "**

**"1. Identify all mentions of specific liver pathology, full terms or abbreviations used in the report, "**

**"for example: primary sclerosing cholangitis (PSC), secondary sclerosing cholangitis (SSC), primary biliary cholangitis (PBC), "**

**"autoimmune hepatitis (AIH), hepatitis, granulomatous hepatitis, overlap syndrome/AIH-PSC, portal vein thrombosis, "**

**"budd-chiari syndrome, cholelithiasis, Non-alcoholic fatty liver disease (NAFLD), drug-induced hepatitis. "**

**"2. Extract the entire sentence where each liver pathology is embedded. "**

**"3. Format all the sentences containing liver pathologies into a single space-separated list. "**

**"4. For each liver pathology found, append ALL rationale from the report ONLY explaining this diagnosis or pathology in curly braces '{}' directly after the liver pathology. "**

**"For example: primary sclerosing cholangitis {PSC, onion skin fibrosis}. "**

**"5. Identify the type of medical scan or test (e.g., MRCP, MRI Liver, Ultrasound Abdomen, liver biopsy) analysed and append this within each set of curly braces identified. "**

**"Specific reference does not need to be made to example pathologies (e.g., PSC) if they are not included in the report. "**

**"6. If no liver pathologies are found within the report file, return 'no liver pathology found'. "**

**"The output should consist of the reformatted text as a single space-separated string. "**

**"The output should therefore read, for example, 'primary sclerosing cholangitis {PSC, onion skin fibrosis, MRCP}'. "**

**"STRICTLY output in the requested format and do not add any extra text."**

**f"\n\nMedical Report:\n{report_text}"**

**)**

A “clinician-in-loop” methodology was employed to quality control the resultant output (Z.G). The extraction of finding context allowed for verification of each pathology. Heterogeneity of findings (i.e. “sclerosing cholangitis” versus “primary sclerosing cholangitis”) was handled by clinician review and structuring output according to overarching cause – i.e. IBD-associated liver disease (PSC, PBD, AIH/PSC overlap). A full list of liver pathology categories, by which findings were categorised can be seen in **SUPPLEMENTARY TABLE A**. All IBD-associated liver disease instances were manually verified using the electronic health records. The number of IBD-associated liver disease cases was compared with the existing gold-standard for case identification, coded electronic record data from the existing database.

| Liver Pathology Category |  | Finding |
| --- | --- | --- |
| Steatosis |  | Hepatic steatosis, alcoholic fatty liver disease |
| Cholelithiasis |  | Cholelithiasis, Gallbladder sludge |
| Structural Malformation |  | hepatic cysts, fundal gallbladder adenomyomatosis, hepatic lesions, Haemangioma, Liver cysts, hepatic adenoma, Biliary cysts, hepatic angiomyolipoma, arteriovenous malformation, Reidel's lobe of the liver, choledochal variant |
| Stone Disease |  | CBD dilatation |
| IBD-associated liver disease |  | Primary sclerosing cholangitis, autoimmune hepatitis, primary biliary cholangitis, AIH/PSC overlap syndrome |
| Cholecystitis |  | Cholecystitis |
| Hepatitis |  | Hepatitis |
| Hepatomegaly |  | Hepatomegaly, Hepatosplenomegaly |
| Cholecystectomy |  | Cholecystectomy |
| Infection |  | Hepatic abscess, cholangitis, Liver abscess |
| Chronic Liver Disease |  | Cirrhosis, liver fibrosis, fibrosis, chronic liver disease, portal hypertension, Liver cirrhosis |
| Cancer |  | hepatocellular carcinoma |
| Cholestasis |  | obstetric cholestasis, Cholestasis |
| *SUPPLEMENTARY TABLE A: A table displaying large-language model extracted clinical findings and liver pathology categorisation* | | |

**SUPPLEMENTARY RESULTS: LARGE-LANGUAGE MODEL OUTPUT**

The LLM output comprised of 670 “liver disease” phrases, including the context from which the relevant pathology or finding was extracted. The electronic record contained 24 coded cases with relevant diagnoses, with n=2 cases not identified by LLM-structuring. These cases’ electronic health records were reviewed, which identified that these were mistakenly coded as having a relevant liver disease diagnosis and were therefore not included in downstream analysis. IBD-associated liver disease diagnoses were identified (n=35) utilising the outlined large-language model, clinician-verified method. Performance of LLM-clinician method compared with routine electronic health record extraction is presented in **SUPPLEMENTARY FIGURE A**.


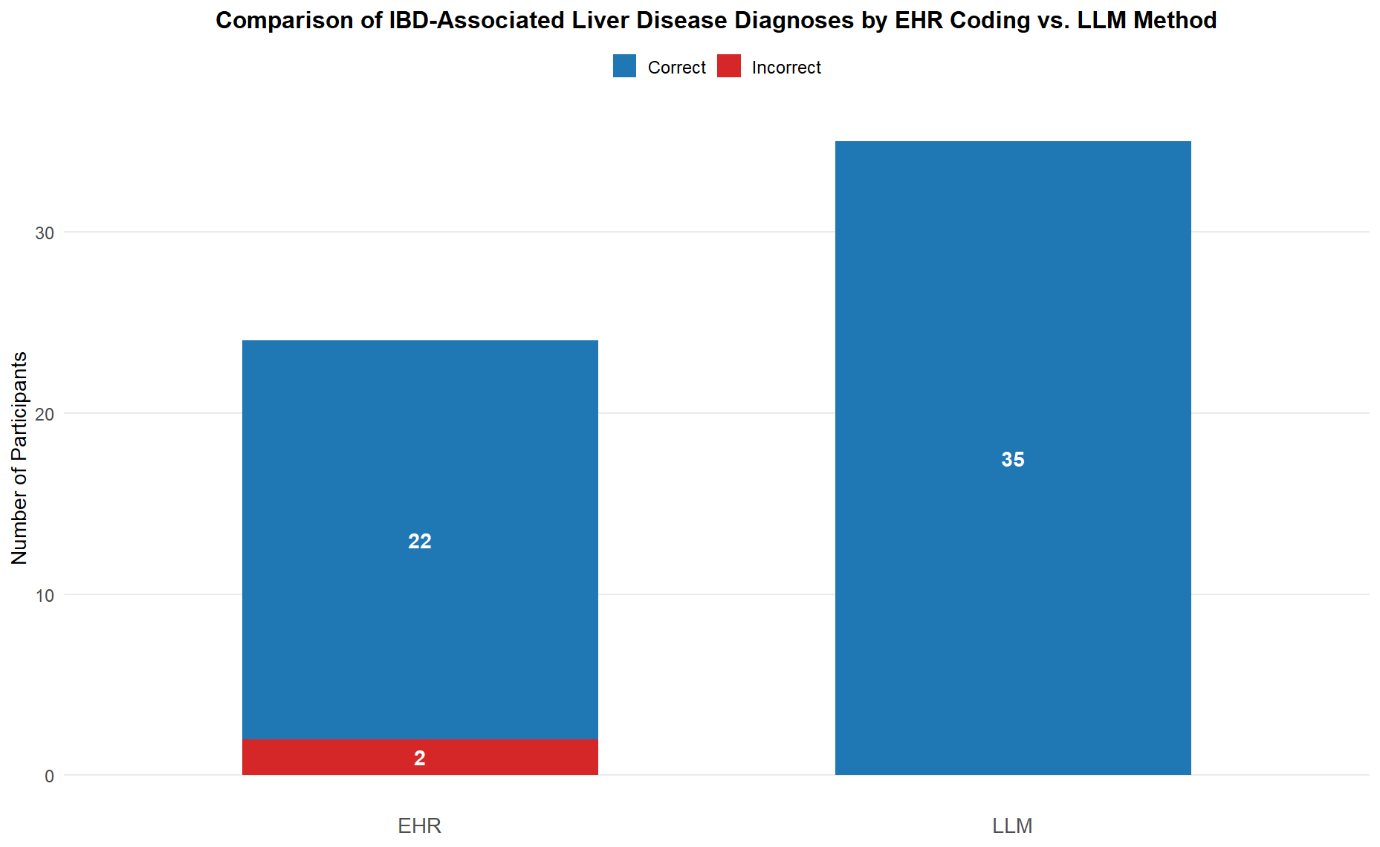


***Supplementary Figure A: A graph demonstrating the number of IBD-associated liver disease (IBDALD) cases identified in electronic health record (EHR) coding compared to Large-language model (LLM) with clinician-in-loop methodology***

1 Pteredactyl PII - a Hugging Face Space by MattStammers. https://huggingface.co/spaces/MattStammers/pteredactyl_PII (accessed 2 May 2025)
